# Multiple Patterns of Perirenal Fat Invasion Are Associated With a Poorer Prognosis Compared With Isolated Invasion: A Proposal for a Revision of T3aN0M0 TNM Staging System

**DOI:** 10.3389/fonc.2020.00336

**Published:** 2020-03-11

**Authors:** Zhixian Wang, Kai Yu, Yunpeng Zhu, Chunxiang Feng, Chang Liu, Shiliang Liu, Jing Wang, Xiaoyong Zeng

**Affiliations:** ^1^Department of Urology, Tongji Medical College, Tongji Hospital, Huazhong University of Science and Technology, Wuhan, China; ^2^Department of Urology, Tongji Medical College, Wuhan Union Hospital, Huazhong University of Science and Technology, Wuhan, China; ^3^Department of Ultrasonography, Tongji Medical College, Tongji Hospital, Huazhong University of Science and Technology, Wuhan, China

**Keywords:** kidney malignant, TNM, perirenal fat, involvement, prognosis, extrarenal, T3a

## Abstract

**Objectives:** Currently, renal cell carcinoma (RCC) presenting with perisinus fat invasion (PSI) and/or perinephric fat invasion (PFI) is merged as one entity, pathological T3a (pT3a); however, the combination of PFI and PSI (PFI+PSI) may not be associated with equivalent prognosis compared with either PFI or PSI alone (PFI/PSI). Here, we analyzed the prognostic significance of PFI+PSI vs. PFI/PSI in pT3aN0–1M0–1 RCC.

**Method:** We identified 5,290 patients with pT3aN0–1M0–1 RCC, treated by nephrectomy, from the Surveillance, Epidemiology and End Results database, between 2010 and 2016. Cox proportional hazards regression and Fine and Gray competing risks regression were fitted to assess risks of survival outcomes, respectively. 1:1 propensity score method was used to minimize differences in the covariates' distributions.

**Results:** Among all patients, 746 patients (14.1%), 2,569 patients (48.5%) and 1,975 patients (37.3%) experienced PFI+SI, PFI, and PSI, respectively, and 3,952 patients (74.7%) without diseases of lymphnode (N1) and/or distant metastasis (M1). PFI alone compared with PSI alone showed a comparable overall survival (OS) and cancer-special survival (CSS), either PFI or PSI alone experienced a better OS and CSS than PFI+PSI. In patients with pT3aN0M0 RCC, PFI+PSI compared with the PFI/PSI was significantly associated with worse OS with hazard ratio (HR) [95% confidence interval (CI)]: 1.38 [1.12–1.69], *p* = 0.002 and 1.41 [1.06–1.87], *p* = 0.017 for unmatched data and matched data, respectively, and higher RCC-special mortality (HR [95%CI]: 1.55 [1.21–1.99], *p* = 0.001 and 1.70 [1.19–2.43], *p* = 0.004 for unmatched data and matched data, respectively). However, in pT3aN1/M1 RCC patients, PFI+PSI was not significantly associated with RCC-special mortality (HR [95%CI]: 1.02 [0.85–1.23], *p* = 0.800 and 0.99 [0.79–1.24], *p* = 0.920 for unmatched data and matched data, respectively) in comparison with PFI/PSI. In addition, invasion type was not an independent risk factor for patient's prognostication in the pT3a RCC with diseases of N1 and/or M1 (all *p* > 0.5).

**Conclusion:** Multiple invasion patterns (PFI+PSI) are associated with inferior survival relative to PFI/PSI alone in patients with pT3aN0M0 RCC; however, these effects are masked in patients with metastatic disease. These results warrant consideration in the development of the next edition of the tumor-node-metastasis staging system, to improve risk stratification.

## Introduction

The American Joint Committee on Cancer (AJCC) published the first Tumor-Node-Metastasis (TNM) staging manual in 1977 (1). Since then, the TNM staging system has become the global standard for the classification of cancer. In late 2016, the 8th edition of the AJCC Cancer Staging Manual was published, with an implementation date of 1st January 2018 for clinical practice and cancer registry reporting. Changes to the staging of kidney cancer were minimal compared with other sites within the urinary and male genital system (1, 2). However, the pathological T3a (pT3a) stage of renal cell carcinoma (RCC) was revised extensively in the 8th edition of the AJCC TNM staging system. In the pT3a category, the word “grossly” is now used to describe the renal vein. Furthermore, “segmental branch extension” was removed and the “muscle-containing branches” were changed to “segmental vein.” In addition, “invasion of the pelvicalyceal system” was added (1, 2).

A major focus of the TNM staging system is the prognostic discrimination between different staging categories. The T stages for RCC are defined by tumor size and local extra-renal invasion. Although the classification of pT3a underwent some major changes in the 8th edition, this stage is defined solely based on anatomical extra-renal extension, regardless of different patterns of invasion. Furthermore, in the primary pT3a tumor staging system, different locations of the extra-renal extension were considered to be associated with a comparable prognosis. Several previous studies have investigated the effects of different sites of fat invasion on prognosis (3–8) and shown that different locations of extrarenal invasion, including renal vein invasion (RVI), perirenal fat invasion (PFI), or perisinus fat invasion (PSI), do not differ significantly from one another with regards to the survival of patients with pT3a RCC. Actually, in our study, we also validated it, PFI alone and PSI alone showed comparable survival outcomes, therefore, merging these three isolated conditions into one stage seems reasonable. However, an increasing body of evidence now indicates that the combined presence of these conditions is associated with worse outcomes compared with each condition alone (3) and suggests that this should be considered when compiling the next edition of the AJCC TNM staging system. Nevertheless, previous studies have been limited by sample size and population characteristics.

In the present study, we aimed to better estimate the impact of the concomitant presence of both PFI and PSI (PFI+PSI) on the prognosis of patients with pT3a RCC, compared with single patterns of PFI or PSI alone (PFI/PSI). Our objective was to provide clinicians with a critical benchmark for counseling prognosis, postoperative follow-up monitoring, and better stratification for patient prognosis. The Surveillance, Epidemiology, and End Results (SEER) database has included the feature of fat invasion beyond the renal capsule since 2010 (https://seer.cancer.gov/). In this study, we used data from the SEER database to evaluate the survival outcomes associated with different patterns of perirenal fat invasion.

## Methods

### Study Population

We searched the SEER-18 registries database for all patients ≥ 18 years of age who had been pathologically diagnosed with pT3a RCC (using the 7th edition of the AJCC TNM staging system) in which the primary tumor site was the renal parenchyma and not the renal-pelvic system (TNM 7/CS v0204+ Schema: Kidney Parenchyma). In all patients, RCC was the only primary malignancy. All patients had undergone partial nephrectomy or radical nephrectomy (RX Summ–Scope Reg LN Sur [2003+]: 30, 40, 50, 70, and 80). Since 2010, the location of invasion beyond the capsule was added to the classification system (CS site-specific factor 1 [2004+ varying by schema]); therefore, the entire study population consisted of patients diagnosed between 2010 and 2016. Patients classified as having tumors with invasion beyond the capsule, with codes 010 (lateral invasion: perinephric fat), 020 (medial invasion: perisinus fat/renal sinus), and 030 (combined 020 and 010), were included in our analysis, whereas those with invasion beyond the capsule, not otherwise specified (991), or unknown or no information (999), were not included. This study was approved by the SEER program managers (Username: 10062-Nov2018) and the institutional review board.

Patients were also excluded if tumor size was > 15 cm, if the information was missing or if the SEER cause of death record stated that a death certificate was unavailable, or was available but with no cause of death recorded. We also excluded patients if the recorded cause of death was unknown, missing, or invalid and if they had been followed-up or died within 1 month of surgical treatment.

### Variables and Outcomes for Analysis

We recorded a range of patient demographic variables, including year of diagnosis, age at diagnosis (years), sex and race (white and other). Tumor variables included histological cell type (clear cell RCC [ccRCC], non-clear cell RCC [nccRCC], or unidentified), tumor grade (Grade I, II, III, and IV), sarcomatoid differentiation (+ or –), type of invasion beyond the capsule in a pT3a tumor (PFI/PSI vs. PFI+PSI), tumor size (mm), regional lymph node involvement (N0 or N1, Nx), and distant metastatic disease (M0 or M1).

The primary outcome of interest was overall survival (OS). The cause of death was determined from the SEER database. Patients who died from RCC were classified as cancer-specific mortality (CSM; in this study, CSM was only due to RCC), and those who died from other causes were designated as competing events before CSM. The duration of survival was defined as the time elapsed from the date of diagnosis to the date of death or last contact.

### Statistical Analyses

Continuous variables are described as mean (standard deviation [SD]) if normally distributed, or median (interquartile range [IQR]) if not normally distributed, and compared with a Student's *t*-test or Wilcoxon rank-sum test, respectively. Categorical variables are presented as frequencies (%) and were compared using χ^2^ tests.

For a better comparison of the two groups (PFI+PSI *vs*. PFI/PSI), 1:1 nearest-neighbor propensity score analysis was then performed to reduce bias due to the non-random occurrence of pT3aN0-1M0-1 RCC with PFI/PSI as opposed to PFI+PSI and PFI vs. PSI. The propensity score is the probability of pT3a RCC presenting a given fat invasion type based on the patient baseline demographics and tumor characteristics. To estimate the propensity of experiencing a invasion type PFI+PSI vs. PFI/PSI and PFI vs. PSI, a logistic regression model was established that included the covariates of year at diagnosis, age at diagnosis, sex, race, tumor size, tumor grade, sarcomatoid features, lymphnode invasion, and distant metastasis with standardized mean difference more than 10% in different groups of pT3aN0-1M0-1 RCC, pT3aN0M0 RCC, and pT3aN1/M1 RCC. This practice can minimize bias and balanced variables difference in the distribution between the PFI+PSI cohort and the PFI/PSI cohort.

The Kaplan–Meier method, with the log-rank statistic, was used to compare overall survival (OS) and cancer-special survival (CSS) between the PFI+PSI and PFI/PSI groups. Multivariate Cox proportional hazards regression models were also fitted to assess the effect of age (continuous), sex (female/male), ethnicity (white *vs*. other), tumor side (right *vs*. left), histological subtype (ccRCC vs. nccRCC and unidentified), FG (III-IV vs. I–II), sarcomatoid differentiation (yes vs. no), tumor size (continuous), nodal status (N_0_ vs. N_1_) and distant metastases (M_0_ vs. M_1_) on all mortality. In these models, we used Cox regression analysis with backward stepwise regression to select variables and identify independent variables associated with all-cause mortality.

Using multivariate Fine and Gray competing risks proportional hazard regression models, we adjusted the pT3a invasion type for other risk variables (age at diagnosis, tumor size, histology type, lymph node status, distant metastasis status, sarcomatoid differentiation, and tumor grade) to predict mortality by RCC and other causes. Subgroup analysis was carried out to further compare pT3a perirenal fat invasion patterns (PFI/PSI vs. PFI+PSI) on all-cause mortality and CSM.

*P* < 0.05 were considered to represent statistically significant differences. All reported P values are two-sided. All analyses were conducted using the R statistical package (v.3.5.2; Foundation for Statistical Computing, Vienna, Austria; https://www.r-project.org).

## Results

The study sample was a pooled cohort of 5,290 patients with histologically confirmed pT3aN0–1M0–1 RCC. A comparison of the demographic and clinicopathological characteristics of unmatched and matched patients is presented in Table 1. Concomitant PFI+PSI was detected in 746 patients (14.1%). Compared with patients diagnosed with invasion of PFI/PSI alone, those with PFI+PSI invasion had a significantly larger tumor size (mean 87.0 vs. 73.7 mm, *p* < 0.001), a significantly greater proportion of grade III-IV tumors (73 64.8%, *p* < 0.001), a significantly higher incidence of sarcomatoid differentiation-positive results (20.4 vs. 11.1%, *p* < 0.001), and a significantly higher incidence of local and metastasis classifications: N_1_ (19.8 vs. 9.4%, *p* < 0.001) and M_1_ (32.0 vs. 16.4%, *p* < 0.001). However, there were no significant differences between the cohort of patients with N1 and/or M1 (Table 1). All the PFI+PSI was matched by PFI/PSI and all co-variables' distribution were made the standardized mean difference <10% between the cohort of PFI+PSI and PFI/PSI (Table 1).

**Table 1:** Clinicopathological characteristics for 5290 pT3a RCC patients.

**Covariables**	**PFI+PSI group**	**Unmatched** **PFI/PSI group**	***P***	**SMD**	**Matched** PFI/PSI group^*^	P^*^	SMD^*^
**pT3aN0–1M0–1 cohort**							
*N*	746	4,544			746		
**Pattern: PFI/PSI (%)**	/	2,569/1,975 (56.5/43.5)	/	/	410/336 (54.9/45.1)	/	/
**Year at diagnosis (%)**			0.280	0.11		0.951	0.07
2010	99 (13.3)	640 (14.1)			90 (12.1)		
2011	81 (10.9)	554 (12.2)			79 (10.6)		
2012	86 (11.5)	611 (13.4)			99 (13.3)		
2013	91 (12.2)	575 (12.7)			94 (12.6)		
2014	132 (17.7)	667 (14.7)			130 (17.4)		
2015	119 (16.0)	695 (15.3)			113 (15.1)		
2016	138 (18.5)	802 (17.6)			141 (18.9)		
**Age at diagnosis. mean (SD)**	63.4 (11.2)	62.6 (11.6)	0.109	0.07	63.3 (11.2)	0.696	0.03
**Race: White/Other (%)**	637/109 (85.4/14.6)	3,934/610 (86.6/13.4)	0.413	0.03	637/109 (85.4/14.6)	1.000	<0.001
**Sex: Female/Male (%)**	196/550 (26.3/73.7)	1,351/3,193 (29.7/70.3)	0.060	0.08	182/564 (24.4/75.6)	0.439	0.04
**Side: Left/Right (%)**	416/330 (55.8/44.2)	2,419/2,125 (53.2/46.8)	0.213	0.05	423/323 (56.7/43.3)	0.754	0.02
**Size. mean (SD)**	87.0 (31.5)	73.7 (31.5)	<0.001	0.42	87.6 (31.9)	0.487	0.04
**Lymphnode**. ***N*** **(%)**			<0.001	0.30		0.952	0.02
N0	580 (77.7)	4,021 (88.5)			580 (77.7)		
N1	148 (19.8)	427 (9.4)			150 (20.1)		
NX	18 (2.4)	96 (2.1)			16 (2.1)		
**Distant metastasis: M0/M1 (%)**	507/239 (68.0/32.0)	3,801/743 (83.6/16.4)	<0.001	0.37	513/233 (68.8/31.2)	0.781	0.02
**Histology: ccRCC/others (%)**	526/220 (70.5/29.5)	3,227/1,317 (71.0/29.0)	0.811	0.01	529/217 (70.9/29.1)	0.909	0.01
**Sarcomatoid: –/+** **(%)**	594/152 (79.6/20.4)	4,041/503 (88.9/11.1)	<0.001	0.26	608/138 (81.5/18.5)	0.395	0.05
**Grade (%)**			<0.001	0.32		0.592	0.07
Grade I	15 (2.0)	141 (3.1)			12 (1.6)		
Grade II	179 (24.0)	1,459 (32.1)			163 (21.8)		
Grade III	285 (38.2)	1,936 (42.6)			307 (41.2)		
Grade IV	267 (35.8)	1,008 (22.2)			264 (35.4)		
**pT3aN0M0 cohort**							
*N*	434	3,518			434		
**Pattern: PFI/PSI (%)**	/	2,026/1,492 (57.5/42.4)	/	/	243/191 (55.9/44.1)	/	/
**Year at diagnosis (%)**			0.687	0.10		0.736	0.13
2010	61 (14.1)	498 (14.2)			59 (13.6)		
2011	54 (12.4)	425 (12.1)			54 (12.4)		
2012	51 (11.8)	478 (13.6)			54 (12.4)		
2013	52 (12.0)	438 (12.5)			59 (13.6)		
2014	76 (17.5)	517 (14.7)			62 (14.3)		
2015	61 (14.1)	551 (15.7)			74 (17.1)		
2016	79 (18.2)	611 (17.4)			72 (16.6)		
**Age at diagnosis. mean (SD)**	64.7 (11.1)	63.1 (11.6)	0.007	0.14	65.0 (11.4)	0.699	0.03
**Race: White/Other (%)**	372/62 (85.7/14.3)	3,049/469 (86.7/13.3)	0.635	0.03	371/63 (85.5/14.5)	1.000	0.01
**Sex: Female/Male (%)**	115/319 (26.5/73.5)	1,067/2,451 (30.3/69.7)	0.112	0.09	119/315 (27.4/72.6)	0.819	0.02
**Side: Left/Right (%)**	239/195 (55.1/44.9)	1,828/1,690 (52.0/48.0)	0.241	0.06	250/184 (57.6/42.4)	0.494	0.05
**Size. mean (SD)**	82.1 (31.6)	69.2 (30.4)	<0.001	0.42	83.0 (31.2)	0.663	0.03
**Histology: ccRCC/others (%)**	325/109 (74.9/25.1)	2,562/956 (72.8/27.2)	0.393	0.05	323/111 (74.4/25.6)	0.938	0.01
**Sarcomatoid: –/+** **(%)**	377/57 (86.9/13.1)	3,281/237 (93.3/6.7)	<0.001	0.22	385/49 (88.7/11.3)	0.468	0.06
**Grade (%)**			<0.001	0.25		0.938	0.04
Grade I	14 (3.2)	130 (3.7)			16 (3.7)		
Grade II	136 (31.3)	1,320 (37.5)			142 (32.7)		
Grade III	171 (39.4)	1,508 (42.9)			164 (37.8)		
Grade IV	113 (26.0)	560 (15.9)			112 (25.8)		
**pT3aN1 or/and M1 cohort**							
*N*	300	951			300		
**Pattern: PFI/PSI (%)**	/	510/441 (63.6/46.4)	/	/	154/146 (51.3/48.7)	/	/
**Year at diagnosis (%)**			0.167	0.20		0.999	0.05
2010	37 (12.3)	136 (14.3)			37 (12.3)		
2011	26 (8.7)	122 (12.8)			24 (8.0)		
2012	35 (11.7)	129 (13.6)			35 (11.7)		
2013	37 (12.3)	129 (13.6)			41 (13.7)		
2014	53 (17.7)	146 (15.4)			54 (18.0)		
2015	56 (18.7)	141 (14.8)			56 (18.7)		
2016	56 (18.7)	148 (15.6)			53 (17.7)		
**Age at diagnosis. mean (SD)**	61.4 (10.9)	60.8 (11.5)	0.512	0.06	62.6 (10.4)	0.661	0.06
**Race: White/Other (%)**	253/47 (84.3/15.7)	821/130 (86.3/13.7)	0.441	0.06	259/41 (86.3/13.7)	0.564	0.06
**Sex: Female/Male (%)**	77/223 (25.7/74.3)	264/687 (27.8/72.2)	0.525	0.05	80/220 (26.7/73.3)	0.853	0.02
**Side: Left/Right (%)**	168/132 (56.0/44.0)	555/396 (58.4/41.6)	0.513	0.05	169/131 (56.3/43.7)	1.000	0.01
**Size. mean (SD)**	94.4 (30.1)	91.1 (29.5)	0.092	0.11	92.8 (29.5)	0.871	0.00
**Lymphnode** ***N*** **(%)**			0.401	0.09		0.665	0.08
N0	146 (48.7)	503 (52.9)			136 (45.3)		
N1	148 (49.3)	427 (44.9)			156 (52.0)		
NX	6 (2.0)	21 (2.2)			8 (2.7)		
**Distant metastasis: M0/M1 (%)**	61/239 (20.3/79.7)	208/743 (21.9/78.1)	0.628	0.04	62/238 (20.7/79.3)	1.000	0.01
**Histology: ccRCC/others (%)**	191/109 (63.7/36.3)	611/340 (64.2/35.8)	0.909	0.01	190/110 (63.3/36.7)	1.000	0.01
**Sarcomatoid: –/+** **(%)**	206/94 (68.7/31.3)	690/261 (72.6/27.4)	0.219	0.09	215/85 (71.7/28.3)	0.475	0.07
**Grade (%)**			0.141	0.17		0.910	0.04
Grade I	0 (0.0)	5 (0.5)			0 (0.0)		
Grade II	41 (13.7)	112 (11.8)			41 (13.7)		
Grade III	107 (35.7)	399 (42.0)			102 (34.0)		
Grade IV	152 (50.7)	435 (45.7)			157 (52.3)		

*RCC, renal cell carcinoma; ccRCC, clear-cell renal cell carcinoma; PFI, perinephric fat invasion; PSI, perisinus fat invasion/renal Sinus; SMD, standardized mean difference; IQR, interquartile range; SD, standard deviation*.

* *All variables in the group of PFI/PSI were 1:1 matched, p value and SMD were assessed after propensity score matching performed*.

During a median follow-up period of 24 months (IQR, 10–46 months), ranging from 1 to 83 months, 3,848 patients (72.7%) in the SEER cohort with pT3aN0–1M0–1 were still alive, while 1,091 (20.6%) had died from RCC and 351 (6.6%) had died due to non-cancer-related causes. Among the 3,952 patients with pT3aN0M0 RCC, 432 (10.9%) were classified as CSM during a median follow-up period of 29.0 months (IQR, 12–52 months). However, 59.1% (739/1,251) of pT3a RCC patients with N1 and/or M1 diagnoses died during a median follow-up period of 13 months, of which 52.4% (655/1,251) were classified as CSM.

**Figure 1:**
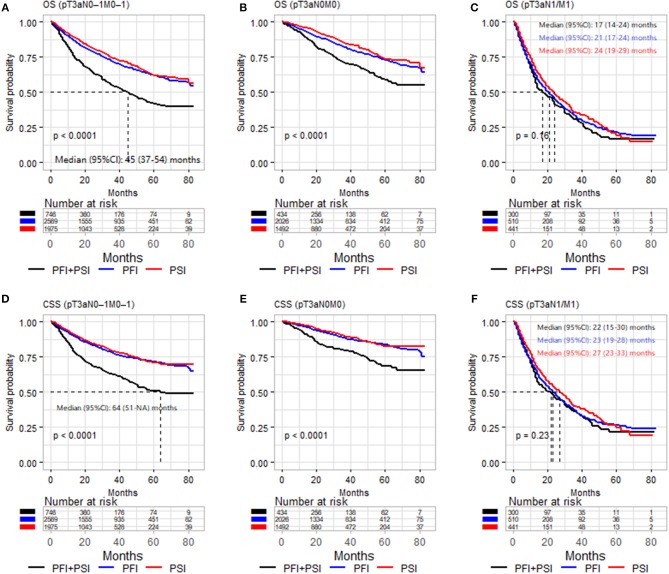
Kaplan–Meier analysis of OS **(A–C)** and CSS **(D–F)** in patients stratified according to pT3a invasion type (PFI vs. PSI vs. PFI+PSI) in the three different populations (pT3aN0–1M0–1, pT3aN0M0, and pT3a N1 and/or M1 RCC cohort). OS, overall survival; CSS, cancer-special survival; PFI, perinephric fat invasion; PSI, perisinus fat invasion/renal Sinus.

Kaplan–Meier analyses were performed in the three different populations: pT3aN0–1M0–1, pT3aN0M0, and pT3a N1 and/or M1 RCC cohort. In order to make sense to merge PFI alone and PSI alone as one classification, we firstly compared the survival outcomes of OS and CSS between PFI alone, PSI alone and PFI+PSI (Figure 1). Figures 1A,D showed PFI alone and PSI alone experienced comparable OS and CSS, and PFI+PSI had worse OS and CSS than bath PFI alone and PSI alone (Figures 1A,B,D,E), however, in the cohort of pT3a RCC with N1 and/or M1 diseases, PFI alone, PSI alone and PFI+PSI showed no significant difference OS (*P* = 0.16) and CSS (*P* = 0.23) compared with each other (Figures 1C,F). And then we furtherly used matched data to analyze PFI *vs*. PSI, since the diagnosis years and age was a significant difference between to group (data was not shown), and which could impact the results. After the year and age were matched, Supplementary Figures 1, 2 still validated the aforementioned results PFI alone and PSI alone experienced a similar prognostication.

**Figure 2:**
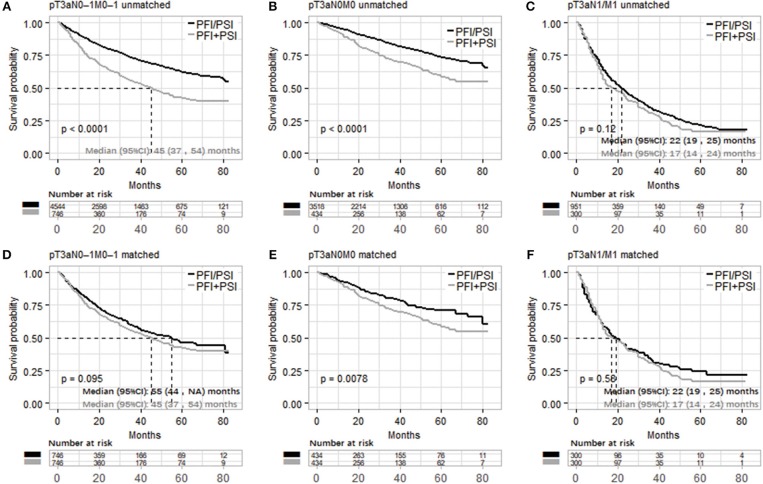
Kaplan–Meier analysis of overall survival in patients stratified according to pT3a invasion type ([PFI/PSI] vs. [PFI+PSI]) in the three different populations (pT3aN0–1M0–1, pT3aN0M0, and pT3a N1 and/or M1 RCC cohort). **(A–C)** and **(D–F)** were unmatched and matched data, respectively. PFI, perinephric fat invasion; PSI, perisinus fat invasion/renal Sinus.

Based on the above results, it's feasible to merge PFI and PSI as one classification, Figure 2 and Supplementary Figure 3 compared OS and CSS between PFI/PSI and PFI+PSI, respectively. Patients with PFI+PSI in the pT3aN0M0 cohort had a lower OS than those with PFI/PSI in both the unmatched and matched cohorts (Figures 2B,E). In the cohort of patients with N1 and/or M1, either the PFI+PSI group or PSI/PFI group showed a worse OS, with a median of 17 and 22 months, respectively (log-rank *p* = 0.12, Figure 2C). Similar results were obtained from the matched cohort of patients with pT3a N1 and/or M1 tumors (log-rank *p* = 0.58, Figure 2F). CSS of PFI/PSI *vs*. PFI+PSI showed the same results (Supplementary Figure 3).

Overall, patients in the PFI+PSI group were at higher risk of both all-mortality (adjusted HR: 1.21; 95%CI: 1.06–1.38, *p* = 0.04) and CSM (adjusted HR: 1.19; 95%CI: 1.01–1.40, *p* = 0.039) in comparison with PFI/PSI (Table 2). In the pT3aN0M0 RCC cohort, PFI+PSI compared with PFI/PSI increased the risk of all-cause mortality by 38% (adjusted HR: 1.38; 95%CI: 1.12–1.69, *p* = 0.02) and that of RCC-caused mortality by 55% (adjusted HR: 1.55; 95% CI: 1.21–1.99, *p* = 0.001). The effects of PFI+PSI *vs*. PFI/PSI on mortality showed no statistical significance in the pT3aN1 and/or M1 cohort. After co-variables were matched, In the pT3aN0-1M0-1 RCC cohort, patients in the PFI+PSI in comparison with PFI/PSI were not significantly associated with both all-mortality (adjusted HR: 1.15; 95%CI: 0.98–1.36, *p* = 0.093) and CSM (adjusted HR: 1.12; 95%CI: 0.92–1.36, *p* = 0.250). However, in the pT3aN0M0 RCC cohort, PFI+PSI increased the risk of all-cause mortality by 41% (adjusted HR: 1.41; 95%CI: 1.06–1.87, *p* = 0.017) and that of RCC-caused mortality by 70% (adjusted HR: 1.70; 95% CI: 1.19–2.43, *p* = 0.04). The effects of PFI+PSI *vs*. PFI/PSI on mortality were not an independent risk factor in the pT3aN1 and/or M1 cohort.

**Table 2:** Multivariate analysis for prediction of outcomes in patients with pT3a RCC.

	**Overall mortality**	***P***	**Mortality by RCC §**	***P***	**Mortality by other §**	***P***
	**HR (95%CI)**		**sHR (95%CI)**		**sHR (95%CI)**	
**Unmatched data**						
**pT3aN0-1M0-1 COHORT**						
Age at diagnosis. per. 1 year	1.03(1.02–1.04)	<0.001	1.01(1.01–1.02)	<0.001	1.06(1.03–1.08)	<0.001
Tumor size, per. 1 mm	1.01(1.00–1.01)	<0.001	1.01(1.01–1.01)	<0.001	1.00(1.00–1.01)	0.300
Lymphnode invasion, N1 (ref. N0)	2.28(1.99–2.60)	<0.001	2.01(1.72–2.36)	<0.001	1.32(0.79–2.20)	0.290
Distant metastasis, M1 (ref. M0)	3.49(3.09–3.93)	<0.001	3.87(3.35–4.48)	<0.001	1.05(0.66–1.70)	0.830
Histology, nccRCC or undefined (ref. ccRCC)	1.29(1.15–1.44)	<0.001	1.29(1.12–1.47)	<0.001	0.99(0.62–1.59)	0.960
Sarcomatoid, + (ref. –)	1.89(1.65–2.16)	<0.001	1.82(1.55–2.15)	<0.001	1.32(0.76–2.28)	0.320
Tumor grade, Grade III+IV (ref. I+II)	1.98(1.71–2.29)	<0.001	2.75(2.27–3.32)	<0.001	0.86(0.54–1.38)	0.540
pT3a invasion type, PFI+PSI (ref. PFI/PSI)	1.21(1.06–1.38)	0.004	1.19(1.01–1.40)	0.039	1.24(0.83–1.85)	0.290
**pT3aN0M0 COHORT**						
Age at diagnosis. per. 1 year	1.04(1.03–1.05)	<0.001	1.02(1.01–1.03)	<0.001	1.07(1.05–1.08)	<0.001
Tumor size, per. 1 mm	1.01(1.01–1.01)	<0.001	1.01(1.01–1.02)	<0.001	1.00(1.00–1.00)	0.970
Histology, nccRCC or undefined (ref. ccRCC)	/	/	1.16(0.94–1.44)	0.160	0.97(0.74–1.28)	0.820
Sarcomatoid, + (ref. –)	2.70(2.18–3.33)	<0.001	2.87(2.21–3.74)	<0.001	1.09(0.65–1.81)	0.750
Tumor grade, Grade III+IV (ref. I+II)	1.92(1.60–2.30)	<0.001	2.99(2.29–3.91)	<0.001	1.09(0.84–1.42)	0.510
pT3a invasion type, PFI+PSI (ref. PFI/PSI)	1.38(1.12–1.69)	0.002	1.55(1.21–1.99)	0.001	1.00(0.68–1.47)	0.990
**pT3aN1 OR/AND M1 COHORT**						
Age at diagnosis. per. 1 year	1.02(1.01–1.02)	<0.001	1.01(1.00–1.01)	0.110	1.08(1.04–1.12)	<0.001
Tumor size, per. 1 mm	/	/	1.00(1.00–1.00)	0.270	1.00(0.99–1.01)	0.940
Lymphnode invasion, N1 (ref. N0)	1.78(1.49–2.11)	<0.001	1.57(1.31–1.89)	<0.001	1.08(0.50–2.33)	0.850
Distant metastasis, M1 (ref. M0)	2.58(2.07–3.21)	<0.001	2.47(1.96–3.12)	<0.001	1.63(0.61–4.33)	0.330
Histology, nccRCC or undefined (ref. ccRCC)	1.46(1.24–1.71)	<0.001	1.31(1.11–1.55)	0.002	1.52(0.69–3.34)	0.300
Sarcomatoid, + (ref. –)	1.53(1.30–1.81)	<0.001	1.46(1.22–1.74)	<0.001	0.93(0.38–2.28)	0.870
Tumor grade, Grade III+IV (ref. I+II)	1.65(1.28–2.12)	<0.001	1.89(1.44–2.48)	<0.001	0.51(0.21–1.22)	0.130
pT3a invasion type, PFI+PSI (ref. PFI/PSI)	/	/	1.02(0.85–1.23)	0.800	1.52(0.76–3.04)	0.230
**Matched data**						
**pT3aN0-1M0-1 COHORT**						
Age at diagnosis. per. 1 year	1.03(1.02–1.04)	<0.001	1.01(1.00–1.02)	0.030	1.06(1.03–1.08)	<0.001
Tumor size, per. 1 mm	1.01(1.00–1.01)	0.001	1.01(1.00–1.01)	0.001	1.00(1.00–1.01)	0.300
Lymphnode invasion, N1 (ref. N0)	2.02(1.66–2.44)	<0.001	1.78(1.43–2.23)	<0.001	1.32(0.79–2.20)	0.290
Distant metastasis, M1 (ref. M0)	3.00(2.49–3.60)	<0.001	3.04(2.46–3.74)	<0.001	1.05(0.66–1.70)	0.830
Histology, nccRCC or undefined (ref. ccRCC)	1.40(1.17–1.67)	<0.001	1.36(1.11–1.66)	0.003	0.99(0.62–1.59)	0.960
Sarcomatoid, + (ref. –)	1.99(1.64–2.41)	<0.001	1.78(1.42–2.23)	<0.001	1.32(0.76–2.28)	0.320
Tumor grade, Grade III+IV (ref. I+II)	2.04(1.56–2.65)	<0.001	2.74(1.98–3.79)	<0.001	0.86(0.54–1.38)	0.540
pT3a invasion type, PFI+PSI (ref. PFI/PSI)	1.15(0.98–1.36)	0.093	1.12(0.92–1.36)	0.250	1.24(0.83–1.85)	0.290
**pT3aN0M0 COHORT**						
Age at diagnosis. per. 1 year	1.05(1.03–1.06)	<0.001	1.03(1.01–1.05)	0.004	1.07(1.05–1.09)	<0.001
Tumor size, per. 1 mm	1.01(1.00–1.01)	0.001	1.01(1.01–1.02)	<0.001	1.00(0.99–1.01)	0.420
Histology, nccRCC or undefined (ref. ccRCC)	1.26(0.92–1.72)	0.146	1.36(0.93–1.99)	0.110	0.91(0.51–1.63)	0.750
Sarcomatoid, + (ref. –)	2.31(1.62–3.29)	<0.001	2.41(1.61–3.61)	<0.001	1.03(0.43–2.50)	0.940
Tumor grade, Grade III+IV (ref. I+II)	2.80(1.90–4.12)	<0.001	6.85(3.49–13.4)	<0.001	1.04(0.61–1.79)	0.880
pT3a invasion type, PFI+PSI (ref. PFI/PSI)	1.41(1.06–1.87)	0.017	1.70(1.19–2.43)	0.004	0.94(0.58–1.54)	0.800
**Unmatched data**						
**pT3aN1 AND/OR M1 COHORT**						
Age at diagnosis. per. 1 year	1.01(1.00–1.02)	0.006	1.00(0.99–1.01)	0.750	1.08(1.04–1.12)	<0.001
Tumor size, per. 1 mm	/	/	1.00(1.00–1.01)	0.530	1.00(0.99–1.01)	0.940
Lymphnode invasion, N1 (ref. N0)	1.59(1.25–2.04)	<0.001	1.46(1.13–1.89)	0.004	1.08(0.50–2.33)	0.850
Distant metastasis, M1 (ref. M0)	2.39(1.73–3.28)	<0.001	2.20(1.59–3.05)	<0.001	1.63(0.61–4.33)	0.330
Histology, nccRCC or undefined (ref. ccRCC)	1.69(1.34–2.13)	<0.001	1.52(1.20–1.94)	0.001	1.52(0.69–3.34)	0.300
Sarcomatoid, + (ref. –)	1.43(1.12–1.82)	0.004	1.36(1.06–1.75)	0.018	0.93(0.38–2.28)	0.870
Tumor grade, Grade III+IV (ref. I+II)	2.01(1.37–2.95)	<0.001	2.64(1.68–4.14)	<0.001	0.51(0.21–1.22)	0.130
pT3a invasion type, PFI+PSI (ref. PFI/PSI)	/	/	0.99(0.79–1.24)	0.920	1.52(0.76–3.04)	0.230

*RCC, renal cell carcinoma; ccRCC, clear-cell renal cell carcinoma; nccRCC, non-clear cell renal cell carcinoma; PFI, perinephric fat invasion; PSI, perisinus fat invasion/renal Sinus; HR, hazard ratio; sHR, Sub-distribution hazard ratio; CI, confidence interval. ¶, Cox proportional hazards regression model, all clinical and pathological features were included in the regression analysis, and the “backward” method for risk variable selection applied. §, Fine and Gray competing risks proportional hazards regression model, variables including age at diagnosis, tumor size, histology type, lymphnode status, distant metastasis status, sarcomatoid differentiation, and tumor grade were used for multivariate analysis*.

**Figure 3:**
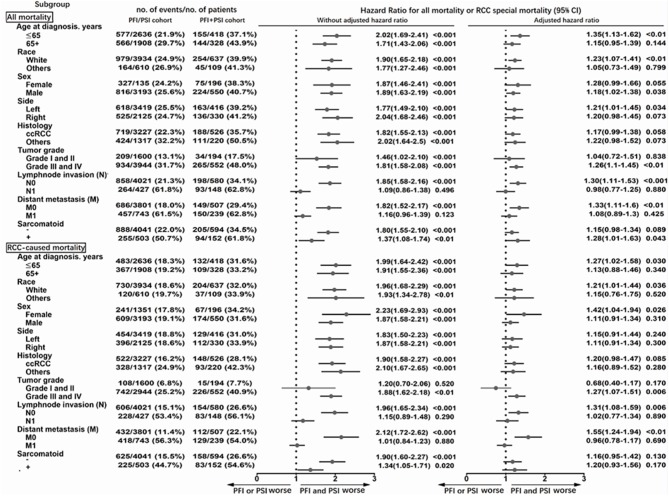
Subgroup analysis of all-cause mortality using the Cox proportional hazards regression model, and mortality from RCC using the Fine and Gray competing risks proportional hazards regression model.

Similar results were obtained from the analysis of subgroups (Figure 3). The effects of PFI+PSI on survival did not differ significantly from those of PFI/PSI of RCC among patients with N1 (adjusted HR for all-mortality: 0.98; 95%CI: 0.77–1.25, *p* = 0.88; adjusted HR for CSM: 1.02; 95%CI: 0.77–1.34, *p* = 0.89) or M1 (adjusted HR for all-mortality: 1.08; 95%CI: 0.89–1.30, *p* = 0.425; adjusted HR for CSM: 0.96; 95%CI: 0.78–1.17, *p* = 0.69) (Figure 2). However, among patients in the N0 cohort and M0 cohort, those with PFI+PSI had a higher risk of all-cause mortality and CSM than those with PFI/PSI, as determined by both univariate and multivariate analyses (Figure 3).

## Discussion

We investigated the prognostic outcomes of patients with pT3a RCC and found that the concomitant presence of PFI+PSI in patients with pT3aN0M0 tumors is associated with significant increases in all-cause mortality and CSM relative to PFI/PSI alone, although this association was not evident in patients with lymph node invasion and/or distant metastasis. These results are consistent with some previously published reports (3–12, 14, 15, 17–19, 21) (Table 3). To improve prognostic stratification, the results of our present study, and those from previous studies, should be considered during the development of the next AJCC TNM staging system.

**Table 3:** Overview of current literature on the prognostic significance of perirenal fat invasion, renal vein involvement and sinus fat invasion in surgically treated pT3 RCC patients.

**Study**	**Study year**	**Number of cohorts (*N*)**	**Study design**	**Results**
Poon et al. (9)	2009	PFI (167) FSI (63)	1. Retrospective single-center study 2. RCC patients (N0-1M0-1) 3. Multivariate analysis predictors	PSI was not an independent predictor of higher RCC-specific mortality in comparison to PFI
Shah et al. (3)	2019	PFI (144) PSI (51) RVI (163) any combination (205)	1. Retrospective single-center study 2. ccRCC patients (Nx/0M0) 3. Multivariate analysis predictors	Any combination of PFI?RVI and PSI was an independent predictor of higher RCC-specific mortality and worse overall survival in comparison to PFI or RVI or PSI alone
Baccos et al. (6)	2013	PFI (63) RVI (59)	1. Retrospective single-center study 2. RCC patients (N0-1M0-1) 3. Multivariate analysis	PFI + RVI was an independent predictor of higher RCC-specific mortality in comparison to PFI or RVI alone
Da costa et al. (5)	2012	PFI (24) RVI (10) PFI+RVI (10)	1. Retrospective single-center study 2. RCC patients (N0-1M0-1) 3. Multivariate analysis	PFI+RVI was an independent predictor of higher RCC-specific mortality and lower disease-free survival in comparison to PFI or RVI alone
Bertini et al. (11)	2009	PFI (70) PSI (12) PFI+PSI (13)	1. Retrospective single-center study 2. ccRCC patients N0-1M0-1 3. Multivariate analysis	PFI+PSI and PSI showed no difference in 5-year RCC-special survival; the presence of PSI is an independent predictor of higher RCC-specific mortality and lower disease-free survival compared to PFI alone in patients with N0M0 diseases
Bertini et al. (14)	2011	PFI or PSI (55) PFI+PSI (26)	1. Retrospective single-center study 2. RCC patients (N0-1M0-1) 3. Kaplan–Meier methods	PFI+PSI showed higher RCC-specific mortality in comparison to PFI or PSI alone
Kresowik et al. (4)	2010	PFI (36) PSI (41) PSI+PFI (33)	1. Retrospective single-center study 2. RCC patients (N0-1M0-1) 3. Multivariate analysis	PFI+PSI was an independent predictor of higher RCC-specific mortality and worse disease-free survival in comparison to PFI or PSI alone
Mouracade et al. (12)	2018	PSI (85) PFI (58)	1. Retrospective single-center study 2. RCC patients (N0M0) 3. Kaplan–Meier methods	PSI did not show higher RCC-specific mortality and worse disease-free survival in comparison to PFI
Margulis et al. (15)	2007	PSI (96) PFI (199) PFI+PSI (70)	1. Retrospective multicenter study 2. RCC patients (N0-1M0-1) 3. Multivariate analysis	Location of extrarenal extension was not an important prognosticator of RCC-specific mortality in the cohort of T3aN0-1M0-1
Bedke et al. (8)	2009	PFI (58) FSI (16) PFI+ FSI (27)	1. Retrospective single-center study 2. RCC patients (N0-1M0-1) 3. Multivariate analysis	Combined PFI + PSI was an independent predictor of higher RCC-specific mortality compared to PFI alone
Kume et al. (17)	2019	PFI (183) RVI+ (158) PFI+RVI (73)	1. Retrospective multicenter study 2. RCC patients (N0M0) 3. Kaplan–Meier methods	PFI+RVI experienced worse RCC-specific mortality compared to PFI or RVI alone
Oh et al. (10)	2018	PFI (124) RVI (40) PFI+RVI (47)	1. Retrospective multicenter study 2. RCC patients (N0M0) 3. Multivariate analysis	PFI+RVI was at an increased risk of recurrence following nephrectomy compared to PFI or RVI alone
Park et al. (18)	2017	PFI (92) RVI (25) PSI (51) PFI+RVI (29) RVI+PFI±PSI (69)	1. Retrospective single-center study 2. RCC patients N0M0 3. Multivariate analysis	Presence of RVI was an independent predictor of worse disease-free survival and higher RCC-specific mortality compared to fat invasion of PFI or PSI
Chen et al. (19)	2017	PFI (94) RVI (20) RVI+PFI (19)	1. Retrospective single-center study 2. RCC patients N0M0 3. Univariate analysis	Patients with RVI had a worse prognosis than those with PFI or PSI
Ball et al. (7)	2016	PFI or PSI (185) main RVI (64) segmental RVI (87)	1. Retrospective single-center study 2. RCC patients N0-1M0-1 3. Multivariate analysis	Main RVI is an independent predictor of worse disease-free survival and higher RCC-specific mortality compared to segmental RVI

*RCC, Renal cell carcinoma; ccRCC, clear cell renal cell carcinoma; PFI, Perirenal fat invasion; RVI, Renal vein involvement; PSI, perisinus fat/renal sinus*.

The last three editions (6th, 7th, and 8th) of the AJCC TNM staging system for RCC all featured minor changes (1), mainly related to the classification of pT3a stage tumors. In the 6th edition, tumors directly invading the ipsilateral adrenal gland and grossly extending into the renal vein, or it's segmental (muscle-containing) branches, were classified as pT3a and pT3b, respectively. This classification underwent a major change in the 7th edition of the AJCC TNM staging system. The changes in the 7th edition included the classification of tumors directly invading the adrenal gland as T4, while those invading the renal vein and its segmental branches were changed to pT3a since RCC tumors with direct ipsilateral adrenal invasion are considered more aggressive than those with just the presence of fat invasion (PFI and/or PSI) (13, 20). In the latest revision (8th edition), the changes mainly involved the inclusion of the renal vein and its tributaries and invasion of the pelvicalyceal system in the pT3a category (2). In addition, due to over-reliance on the anatomical gross inspection of the hilar vessels, tumor extension to renal vessels was commonly missed when using the guidelines described in the 7th edition (2). Consequently, the word “grossly,” previously used to describe the renal vein and segmental branch extension, was removed and “muscle-containing” was changed to “segmental vein” (1, 2).

Numerous studies have investigated the effect of different types of extra-renal invasion on prognosis. For example, Kresowik et al. (4) found no significant difference between PFI alone and PSI alone (HR: 1.62; 95% CI: 0.68–3.90; *p* = 0.279, for PNFI vs. PSFI) and observed a worse prognosis in patients with pT3a tumors with combined PFI+PSI (HR: 2.67; 95% CI: 1.19–5.97; *p* < 0.001). Bedke et al. (8) reported similar results in a series of 106 patients with pT3a, including 54.7% with PFI alone, 19.8% with PSI and 25.5% with PFI+PSI; those with combined invasion had a 2.75-fold higher risk of CSM. Furthermore, in patients with ccRCC, the risk increased to 3.41-fold compared with either PFI or PSI alone. In another study, da Costa et al. (5) investigated a small series of 46 pT3a patients, 52.1% of whom had tumors with fat invasion, 23.9% with RVI, and 23.9% with both fat invasion and RVI; results demonstrated that patients with both fat invasion and RVI exhibited inferior survival relative to those with either of these conditions alone (HR for 5-year progression-free survival: 2.5; 95% CI: 1.014–6.352; *p* = 0.04; HR for CSS: 2.7; 95% CI: 1.07–6.85; *p* = 0.04).

More recently, Shah et al. (3) performed a similar study investigating the prognosis associated with different sites of extra-renal extension in pT3a RCC and also found that any form of combined invasion, including PFI, PSI, and RVI, was associated with a worse prognosis than each alone. These authors further demonstrated significant associations with increased CSM (HR: 1.64; 95% CI, 1.27–2.12; *p* < 0.001) and disease progression (HR: 1.31; 95% CI: 1.04–1.65; *p* = 0.02). In addition, these authors found that there were no significant differences in survival outcomes when compared between patients with isolated PFI, PSI, or RVI invasion. Furthermore, RVI and PSI alone were not significantly associated with disease progression (HR: 0.87; 95 %CI: 0.64–1.19; HR: 0.90; 95% CI: 0.55–1.47, respectively) or DSS (HR: 0.76; 95% CI: 0.53–1.09; HR: 0.81; 95% CI: 0.44–1.49, respectively) relative to PFI alone. Our study was inspired by Shah et al.'s study and our own clinical practice based on the background of the 8th edition RCC TNM staging system. The study furtherly validated the results of Shah et al. study. In our study we found that PFI+PSI is associated with inferior survival relative to PFI/PSI alone in patients with non-metastatic pT3a RCC, this result was inconsistent with Shah et al. study; however, these effects are masked in patients with metastatic disease (N1 or/and M1), which was the absence of Shah et al. study. Therefore, risk stratification should not only be based on fat invasion type but also a consideration of lymphnode invasion and metastasis diseases.

The inconsistent and somewhat controversial conclusions of some previous studies may be attributable to a lack of population homogeneity and small sample size. For example, Thompson et al. (16) investigated a cohort of 212 patients with pT3a disease at the Mayo Clinic Center and found that patients with PSI had an inferior prognosis relative to those with PFI alone. However, Shah et al. (22), based in the same institute, re-assessed these results and found no significant difference in survival between patients with these two separate invasion parameters after excluding patients with metastatic diseases. In another study, Bertini et al. (11) evaluated 105 patients with pT3a ccRCC and found that tumors with PSI had a significant effect on CSM in patients without N_1_/M_1_ compared with PFI alone; however, PSI was not significantly associated with poor CSM in cases with metastatic disease. In contrast, another study, conducted by Margulis et al. (15), included 365 pT3a RCC patients from the M.D. Anderson Cancer Center and found that PSI and PFI were comparable prognostic indicators of CSM in surgically treated patients with pT3a RCC. Furthermore, these authors did not find that patients with PSI+PFI had inferior 5-year survival relative to either parameter alone. Notably, Margulis et al. reported that a higher number of patients with PSI alone (26.3%) and PFI+PSI (55.7%) had metastases in their study; this may have confounded the impact of extrarenal extension patterns on CSM in their multivariate analysis.

In the current TNM staging system, the classification of pT3a only includes tumors extending into the renal vein or its branches; microscopic wall involvement is not mentioned in this particular category. Notably, Park et al. (18) found that pT3aN0M0 patients who presented with RVI had a significantly worse prognosis than those with fat invasion; the 5-year recurrence-free survival and 5-year DSS for RVI/RVI+PFI±PSI vs. PFI alone/PSI alone/PFI+PSI were 33.8 vs. 67.9% (*p* < 0.001) and 63.0 vs. 88.2% (*p* < 0.001), respectively. However, it is important to note that in the RVI/RVI+PFI±PSI group, 52.1% of patients presented with vein-wall invasion and that renal vein-wall involvement was a good predictor of a poor prognosis. Furthermore, RVI presenting simultaneously with vein-wall involvement was shown to significantly increase the risk of CSM (HR: 2.771; *p* = 0.03). Furthermore, positive surgical margins were associated with a markedly higher risk of CSM (HR: 9.462; *p* < 0.001) compared with thrombi only. Differences in the extent of RVI were further investigated by Ball et al. (7), who reported that RVI was generally independently associated with an inferior prognosis relative to segmental RVI.

Collectively, these results indicate that it was reasonable to merge the isolated presence of PFI, PSI, or RVI as a single pT3a classification in the 8th edition of the AJCC TNM staging system. However, we recommend that the concomitant presence of these different types of invasion should be recategorized in the next edition. In the present study, we found that the effects of PFI+PSI compared with PFI/PSI on survival outcomes can be masked by the effects of N1 and M1 (Figure 3). Thus, these results would only be applicable for patients with pT3a RCC without any indication of lymph node or distant progression.

Although the present analysis was robust, the study had some limitations which need to be considered. As the included data were from the SEER database, the retrospective nature of this study was an inherent limitation. Moreover, details regarding symptoms at diagnosis, comorbidity, histopathological features of tumor necrosis and lymph-vascular invasion, the feature of RVI, metastatic patterns, the lack of centralized pathological review, the time of recurrence and the treatment of recurrent disease were not available and hence not analyzed. Despite its limitations, our findings are of significance. Further studies are now required to better inform the future modification of the TNM staging system to improve prognostic discrimination.

## Conclusion

In the present study, we confirmed that the concomitant presence of PFI+PSI was associated with worse survival outcomes compared with PFI/PSI alone in a population of pT3aN0M0 patients but not those with N_1_ and/or M_1_. This finding is not considered in the latest (8th) edition of the AJCC TNM staging system. However, if our findings can be validated further, we recommend that patients with pT3aN0M0 RCC should undergo separate risk stratification for improved prognostic prediction.

## Data Availability Statement

SEER-18 registries database (https://seer.cancer.gov/), data was downloaded from SEER^*^Stat software (Username: 10062-Nov2018).

## Author Contributions

ZW and XZ conceived the study. ZW, KY, and YZ carried out the statistical analyses and drafted the manuscript. ZW and KY interpreted the data. CF, CL, SL, and XZ critically revised the manuscript. All authors have reviewed the final version of the manuscript and approve it for publication and participated in the analysis and interpretation of data.

### Conflict of Interest

The authors declare that the research was conducted in the absence of any commercial or financial relationships that could be construed as a potential conflict of interest.
