# Supplementary material for: Multiple Patterns of Perirenal Fat Invasion Are Associated With a Poorer Prognosis Compared With Isolated Invasion: A Proposal for a Revision of T3aN0M0 TNM Staging System
Source: Front Oncol. 2020 Mar 11;10:336. doi: 10.3389/fonc.2020.00336 (PMC7078176; doi:10.3389/fonc.2020.00336)
Supplement: Supplementary file 1 [file Image_1.pdf]

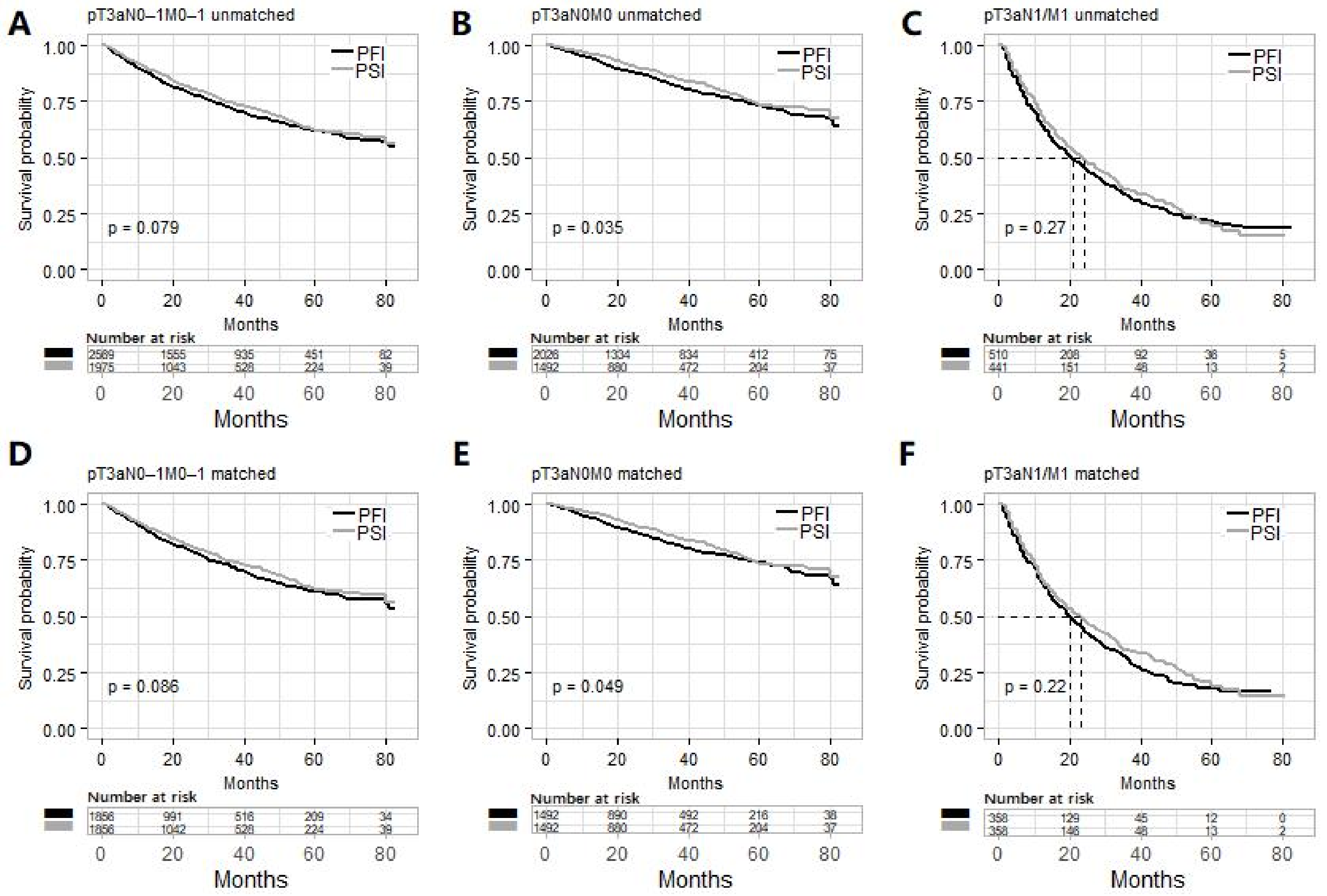

Supplementary figure 1. Kaplan–Meier analysis of overall survival in patients stratified according to pT3a invasion type (PFI *vs*. PSI) in the three different populations (pT3aN0–1M0–1, pT3aN0M0, and pT3a N1 and/or M1 RCC cohort) . Abbreviations: PFI, perinephric fat invasion; PSI, perisinus fat invasion/renal Sinus.

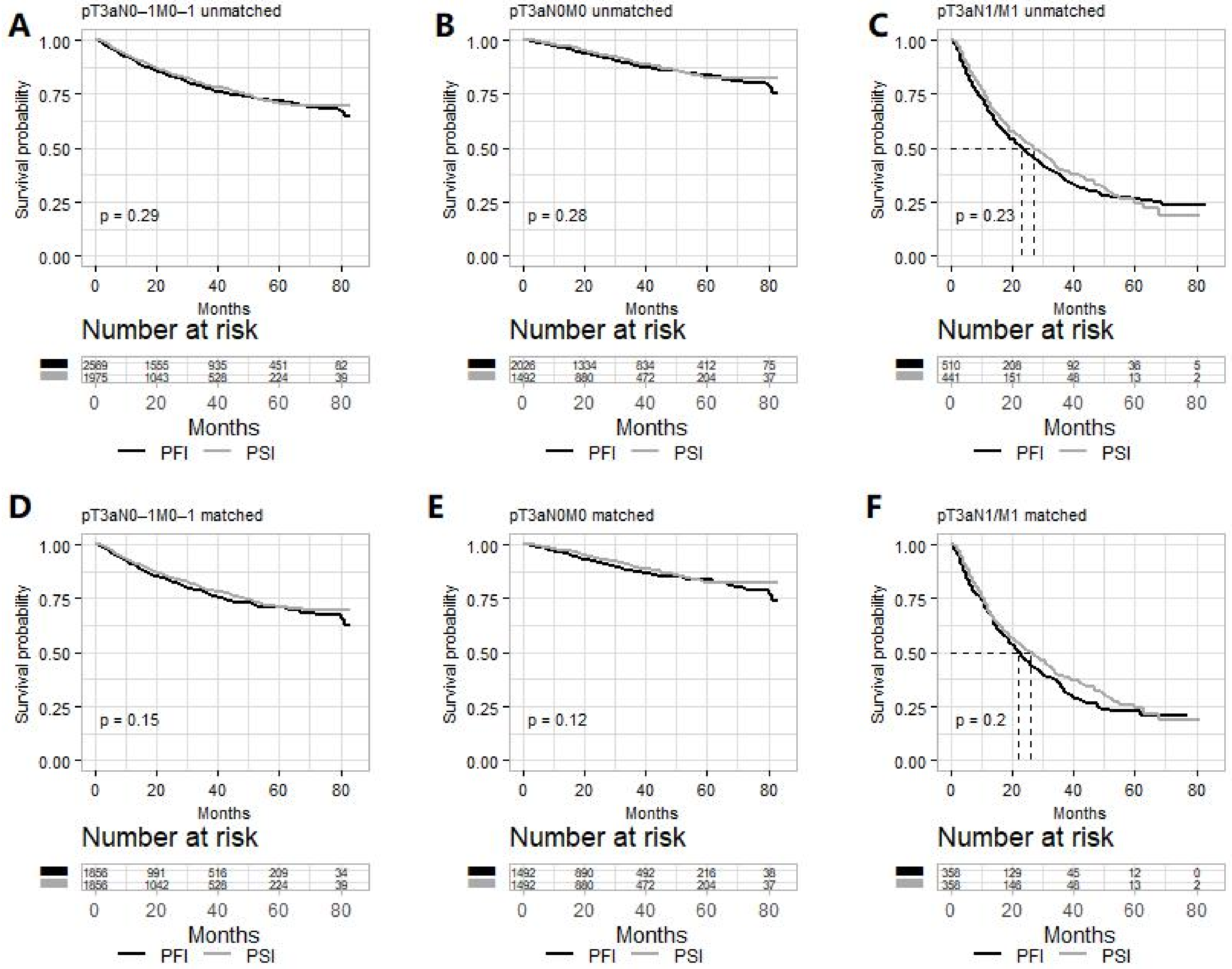

Supplementary figure 2. Kaplan–Meier analysis of RCC-special survival in patients stratified according to pT3a invasion type (PFI *vs*. PSI) in the three different populations (pT3aN0–1M0–1, pT3aN0M0, and pT3a N1 and/or M1 RCC cohort). Abbreviations: RCC, renal cell carcinoma; PFI, perinephric fat invasion; PSI, perisinus fat invasion/renal Sinus.

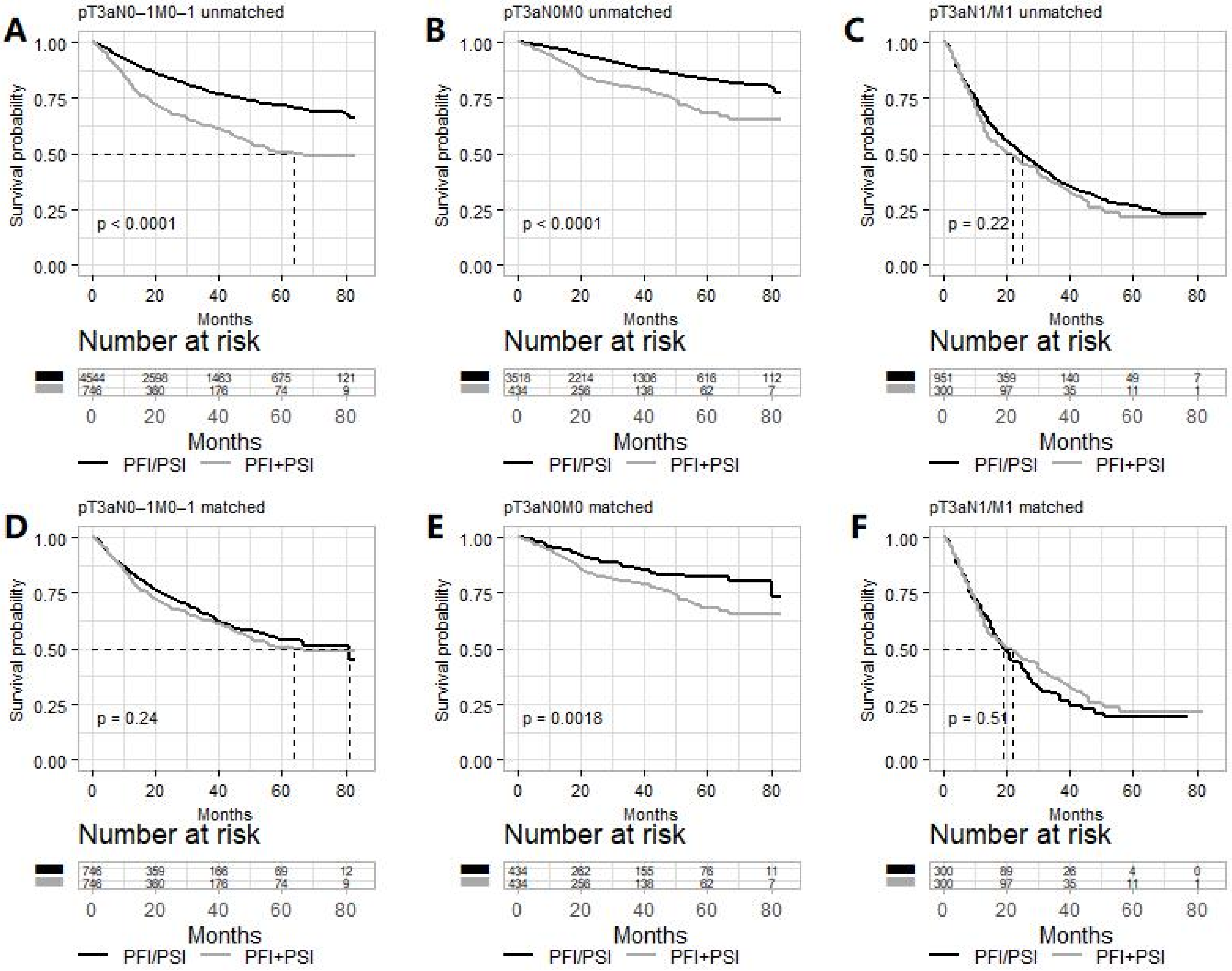

Supplementary figure 3. Kaplan–Meier analysis of RCC-special survival in patients stratified according to pT3a invasion type ([PFI/PSI] *vs*. [PFI+PSI]) in the three different populations (pT3aN0–1M0–1, pT3aN0M0, and pT3a N1 and/or M1 RCC cohort). Abbreviations: RCC, renal cell carcinoma; PFI, perinephric fat invasion; PSI, perisinus fat invasion/renal Sinus.
